# A Short Dissertation on “Ghosts”

**Published:** 1901-05-15

**Authors:** Burt Abell


### A SHORT DISSERTATION ON "GHOSTS."

BY BURT ABELL, D.D.S.

Read before the Southwestern Michigan Dental Society, April 10, 1901.

Ghosts? Why, yes; we've all seen them. They began appearing to us in our boyhood days, after we had been in the jam pot, or stoned somebody's windows, and we've seen them all along the way at every milestone. They come trooping in at night to disturb our dreams; they walk beside us by day, and we see them and wonder if they are visible to any one else.

When we went to college we hoped to leave them behind, but there they were, a legacy of the classes of previous years. As we look back upon them, how trivial they seem, yet how real they were then. What a spook, haunting even our waking moments, was our first examination! Then those first fillings and plates—"May we never meet the shades of 'em."

Our troubles did not end when we began in our own office. You've seen that ghost that walks in in broad daylight and speaks something like this: "I've come back. Yes, I have. I haven't slept for three nights. I knew 'twould ache. I wanted you to pull it in the first place. My uncle's cousin's brother had one filled at Dr. Blank's and it never hurt him a bit.'' (Patient in the chair and one or two in the waiting-room are hearing it all.) Oh! shades of my departing reputation. Mentally you are tearing your hair and thinking "This is what I get for trying to educate the public to save their teeth,'' and you would like to make a ghost of the man who advocates it. In vain you try to explain that it sometimes happens even after one has done his best; that there was no exposure of the pulp; tooth better if pulp is saved; took some chance to save it; couldn't quite tell condition of pulp from her description of case; pulp probably dying and causing pressure; could fix it, etc., etc. Nothing you may say can ever restore her confidence in your professional ability—providing she ever had any. Seriously, does it pay, each and every time, to try to persuade people to preserve their teeth when they demand extraction? Time and again the charge of mercenary motive will be laid at your door. How the righteousness seeps out of us, as with an air of martyred innocence we assure them that the salvation of teeth has become a passion with us. Our business is to save teeth, not to waste them. Inconvenient as a conscience is, what less can we really do than cultivate it in this direction? There is one hope for the future that I trust may prove no shadow but materialize right speedily, and that is, some step toward educating in our public schools the rising generation to care for their teeth. In Canada and many European countries children with decay or disease in the mouth can not enter school. Why isn't it just about as necessary to appoint a dentist to give instruction in dental matters in our public schools as to appoint a health officer in a city? The town board or council can do one as easily as the other, were it only a law.

The argument is raised that the health officer is an agent to prevent the spread of contagious diseases. From a dental standpoint it is getting at the root of the matter to purify the gateway of the human citadel. Our point of attack, then, is the lawmakers, and who shall do it if not the dentists of our land through our associations? If I am not mistaken the matter has been tried; but let us try again, and yet again, until it is accomplished. We have not lived up to our fullest privilege till we have used our best endeavor to make this old world of ours a better place for our having lived in it.

The ghost of departed opportunities is the one that causes us more unhappy moments, perhaps, than any other. He isn't exactly a professional ghost, for we have seen him haunt men of other walks in life. After years of silence we hear of some brother who started in business after we did, perhaps; he has done well financially, bought him a store building, owns his own house, got something in the bank, so report says. "Why, that beats my pile!" we exclaim, and we begin to wonder what we have done with all the shekels that have passed through our hands. Not many stick to the most of us, do they? We inquire a little more closely and we find our brother was willing to do *without* things he did not absolutely need. What a vast difference there is between our *wants* and our *needs*, when we come to think of it. So few professional men make good financiers. Isn't it worth while to study our own case, compare it with more successful ones, in a financial way, and see wherein we may improve, and save, and lay the terrible phantom of Debt that shadows the path of so many of our profession to-day. The Good Book tells us to "covet earnestly the best gifts," and I take it that means among other things to seek the ability to get honestly and save what we get.

Another ghost that comes into our office is the rubber ghost that grins at us from behind the white bars of his castle, and as he rattles his portcullis up and down and steps out, we know our plate was not a success. "It won't stick; it rocks; it comes down," etc., etc. "Make it over? Oh, yes; glad to make our work good." (Oh, what deep joy!) We go at it shaking in our boots, for fear it will be no better next time. *Now*, let's do it right. We take just the best impression we know how, pour our cast, put on our baseplate, flask, pack and vulcanize. What have we got? A plate without teeth that, if the cast was properly carved and beaded, will stick as no plate made in the old way ever thought of sticking. *Now*, we take our bite, set up our teeth, try in the mouth to correct expression, flask, pack and vulcanize. "Lots of trouble?" Oh, yes; more work, more visits of patient, but we know "where we're at" every step of the way. The patient is happy, and that ghost is laid.

But there is another ghost. (We've got 'em, you see.) He appears to us around the borders of fillings. Now, don't feel hurt, brethren. It's somebody else's fillings I mean. We think right off that a prescription of extension for retention will many times lay *him*. But not always. Once in a while we excavate carefully, get good, clear borders, and fill properly, but still there is recurrence of decay. I believe that in such cases we have not excavated beyond the zone affected by the ptomaine, acid, or whatever it is that, confined in the tubuli, has been causing the trouble. The rational procedure, then, is to neutralize that acid with caustic potash or some strong alkali before filling. I am not theorizing on this, but believe I have demonstrated in actual practice that this is right. I remember one case where a brother dentist had put in two sets of fillings in the same teeth, so to speak, and I had refilled the teeth and lost my fillings; I treated then as indicated above, and, after five years, the fillings are there now.

The last ghost I want to speak about is the one that lives up the blind alley called Root Canal. We've tried to lay him with the best medicaments we know of, got his house all sweet and clean and plugged him in tight, and we flatter ourselves he hasn't a ghost of a chance to get out. He does lie still for a year or two, and we begin to feel easy about him, till we read J. Foster Flagg's statement that nine out of ten canals so treated will give trouble in twenty years. *Twenty years*, is it? The horror is back upon us and we begin to ask ourselves *why* we filled that canal at all. The dentin in a living tooth is nourished by both pulp and peridental membrane. When the pulp goes it necessarily follows that the peridental membrane has all of the work to do, and unless the pulp chamber can be kept in a healthy condition the integrity of the dentin can not be preserved. To this end we disinfect and do our best to seal up the apical foramen and lock out infection. We must bear in mind that unless asepsis can be *preserved* the already overworked peridental membrane will rebel and we shall have trouble.

The question of *how* now presents itself, and every man here has his own way and thinks it is the best.

If we follow all that is said on this subject in our dental literature, we will see that we are getting out of the realm of empiricism into that of rationalism in this matter. Time and again we have opened into pulpless teeth and found the canal empty and the tooth quiescent.

Nature has been trying to tell us one of her secrets. I'm not sure we have heard aright, but this much we know: she demands cleanliness. We may preserve this asepsis by mechanical means, as with gutta-percha or some of the metals. But in view of the difficulty of entire extirpation of the filaments at the apex, shall we not be excused if we rely on chemical means as well? Miller, with bi-chlorid of mercury; Boennecken, with formaldeheyd; Wald, with his chemico-metallic, and Soderberg with his mummification, are all trying for the same thing. Which of these is the most feasible to eliminate this skeleton in the closet in any given case only our best judgment and experience can determine; and then—well, *time* is the *test*, after all.

I've worn your patience almost to a shadow with my ghosts, and yet have only touched upon each point, any one of which is suggestive of material enough for a paper. Yet I hope to be as fortunate as the old man in the story, whose son had fired a number of times at a squirrel and missed him every time. The old man's patience could stand it no longer, so taking the gun and wobblingly pointing it at the tree, fired and brought down the squirrel. "There, that's the way to do it," said he. "Well," the son retorted, "you ought to get him. You shot all over the tree." If in shooting all over the tree we can bag one point and get help from it, I shall be satisfied with my staggering effort.

